# A comparison of two anesthesia methods for the surgical removal of maxillary third molars: PSA nerve block technique vs. local infiltration technique

**DOI:** 10.4317/jced.51199

**Published:** 2014-02-01

**Authors:** Ra´ed MA. Al-Delayme

**Affiliations:** 1 B.D.S, S.OMFS.S, C.A.B.OMFS, M.F.D. R.C.S. I., M.O.M.S. R.C.P.S .G, F.F.D (OSOM) R.C.S.I. Dean of The Faculty of Dentistry, Dijla University College, Baghdad, Iraq; 2 B.D.S, S.OMFS.S, C.A.B.OMFS, M.F.D. R.C.S. I., M.O.M.S. R.C.P.S .G, F.F.D (OSOM) R.C.S. I. Senior Specialist at Oral and Maxillofacial Surgery Dept., AL-Yarmuk Teaching Hospital, Baghdad, Iraq

## Abstract

Objectives: The purpose of this study was to compare the effect of PSA block injection with infiltration technique regarding local anesthesia for surgical extraction of upper third molar. Material and Methods: A prospective, intra individual, single-blind randomized controlled trial was designed to study the severity of pain during injection and after surgical extraction of the bilaterally and symmetrically similar upper third molar in a total of 53 patients, in addition to evaluating the need to repeat the injection and requirement of post operative anti-inflammatory tablets. Result: Although the average pain score for all studied times in PSA side was lower than the average pain score in infiltration technique, repeated statistical measures demonstrated that no significant pain reduction occurred in the two techniques. Conclusion: The both tested methods have the same statistic equivalence for the surgical extraction of maxillary third molars.

** Key words:**Surgical extraction, maxillary third molars, PSA block, infiltration.

## Introduction

Surgical extraction of impacted teeth can be either uneventful and uncomplicated, or difficult, with considerable postoperative pain. (1) Maxillary third molars are frequently amenable to removal surgically under local anesthesia (2) .Fear of a dental injection and postoperative pain can prevent patients from seeking dental care and often this fear is related to the feeling of needle penetration and pain during the injection (3). Local anesthesia plays an essential role in making dental treatment comfortable .Also it has been called the most important drug in dentistry. Conversely, local anesthetic injections are seen by many patients as stressful and a reason for avoiding dental treatment (4).

A range of local anesthetic drugs have been used in dentistry among which lidocaine is the most popular (5). The common techniques for providing anesthesia in maxillary molars include posterior superior alveolar (PSA) nerve block and infiltration anesthesia (6).

The majority of the recently published articles evaluate the anesthetic efficacy of the PSA nerve block and maxillary infiltrations either in inflamed pulps or in the normal tooth extraction (6-8). To the knowledge of the author, there is no published data evaluated and compared in terms of the severity of pain during injection and after the surgical extraction of upper third molar, and the need to repeat the injection and requirement of anti-inflammatory tablets.

## Material and Methods

- Data Sampling

This prospective, intra individual, single-blind, randomized controlled trial study was undertaken between September 2009 and June 2010 at the Department of Oral and Maxillofacial Surgery, College of Dentistry, SIUST University. A total of 53 patients, 31(58.4%) males and 22(41.5%) females who underwent a surgical removal of symmetrically bilaterally impacted upper third molars due to prophylaxis or orthodontic purpose with a mean age of 20. 4 ± 3 at the time of surgery, ranged between 17 and 26 years. All the patients who were healthy and non-Smokers having no medications or oral contraceptives in the preoperative period and were free from active local inflammatory lesions, were included in the analysis. The study design was approved by the Research and Ethics Committees of University. All patients were informed as to the nature of the surgical and experimental procedures, and informed consent being obtained before surgery.

An orthopantomographic images were used to ensure the symmetry of the type of impaction and to classify all the impacted maxillary third molars into mesioangular 27 (50.9%), vertical 15(28.3%) and distoangular 11(20.7%) impactions based on Winter`s classification (9) and all teeth were either class B 39(73.5%) or C 14 (26.4%) according to Pell and Gregory classification (10). In all the cases there is more than 2 mm of bone between the impacted maxillary 3rd molar and the maxillary sinus.

- Procedures

Each of the 53 patients was scheduled to undergo bilaterally and symmetrically identical upper third molar surgical extraction (and thus presenting similar surgical extraction difficulty).The two extractions were performed in two separate sessions approximately 4 weeks apart to allow for total recovery from the first one.

In each patient, the choice of which anesthetic techniques were going to be administered, the PSA block technique and on the contra lateral the infiltration technique, was made randomly. The palatal injection was combined to both techniques. A topical anesthetic gel 5% lidocaine (Xylonor Gel, Septodent, U.K.) was placed with a cotton tip applicator. After reaching the target area, aspiration was performed several times during the administration of the injection using standard dental aspirating syringe (KlS, Martin, Germany) fitted with a 25-gauge, long, 0.40×35mm needle (Sterinject, Dentsply, France). The technique of the PSA block was identical to Malamed`s text (11). In the infiltration technique, after two minutes of buccal infiltration, a palatal infiltration was administered. A 1.8 mL of 2% lidocaine hydrochloride with 1:80,000 adrenaline solution (Lingospan special, Septodent, France) was deposited at a rate of 1 mL/min. A second or third injection was given to the patient who has experienced an additional pain. After 5 minutes of the injection of a determined dose of local anesthesia, the surgical procedure was performed.

The surgical procedure was similar in all cases and was performed by the same surgeon using a standardized technique under local anesthesia without any kind of sedation (oral, nasal or venous); full envelop mucoperiosteal flaps were elevated prior to the removal of the third molars. Alveolotomy was carried out using a bur under a concomitant continuous spray of sterile saline solution; the flaps are sutured with a 4-0 silk suture.

After surgery, all of the patients received an oral non-steroidal anti-inflammatory drug (600 milligrams of Ibuprofen every 4-6 hours for four days) and topical chlorhexidine digluconate for seven days .The suture material was removed after one week. All surgical details were noted in a pre-made questionnaire

- Pain Measurement

**Figure 1:**
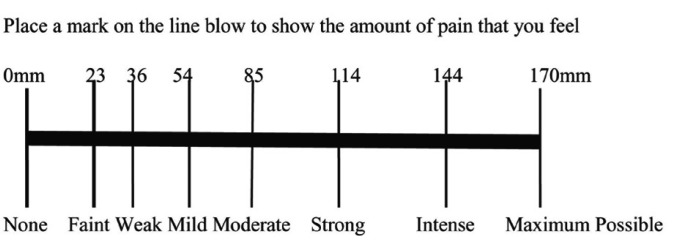
Heft-Parker visual analog scale (VAS) used for assessment of pain.The millimeter demarcations were not shown on the patient`s VAS.

Preoperative pain assessed by a single blind professional operator was different from the surgeon who performed the surgery, repeating each record three times on each case: during the injection, at the end of operation and after 15 minutes from the end of operation, using a 170-mm Heft-Parker visual analog scale (VAS; Fig. 1). Before being administered local anesthetic agent, each patient was given a thorough explanation of the VAS which was divided into 4 categories: no pain corresponded to 0 mm; mild pain was defined as greater than 0 mm and less than or equal to 54 mm and included the descriptors of faint, weak, and mild pain; moderate pain was defined as greater than 54 mm and less than 114 mm; severe pain was defined as equal to or greater than 114 mm and included the descriptors of strong, intense, and maximum possible. There is no strong or intense pain recorded at the end of operation.

- Statistical Analysis

Statistics was performed using the SPSS for Windows (version 13.0, SPSS Inc, Chicago, IL) statistical software package. The pain VAS scores were analyzed by analysis of variance (ANOVA) for repeated measures. X2 were used for significance of age, sex and operation time on the severity of pain .Probability less than 0.05 was considered statistically significant.

## Results

In a total of 53 patients participated in this study, there was no significant correlation between age and sex with the intensity of pain.

The average duration of the surgical procedure starting from the flap reflection to the end of suturing on the PSA side, was 7.83± (4.18) minutes (range, 3-11 minutes); while on the infiltration side, it was 8.47± (4.38) minutes (range, 4-11 minutes), the difference was statistically significant F=21.701 (P<0.01) with intensity of pain. Evaluations at injection showed a 1 (1.8%) positive aspiration with PSA block and without any positive aspiration with infiltration technique.

**Figure 2:**
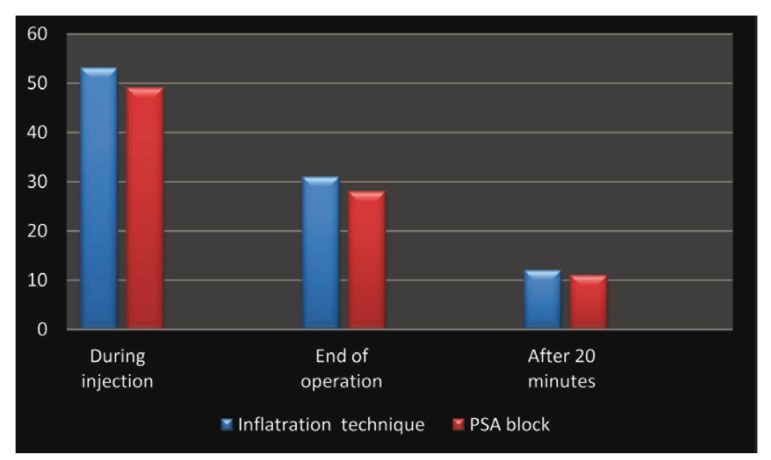
The mean of pain intensity (mm) measured by VSA in two sides.

Although the average pain score for all studied times in PSA side, was lower than the average pain score in infiltration technique, repeated-measures ANOVA demonstrated no significant difference in pain reduction between the two techniques (P > 0.05,  Table 1, Fig. 2).

**Table 1:**
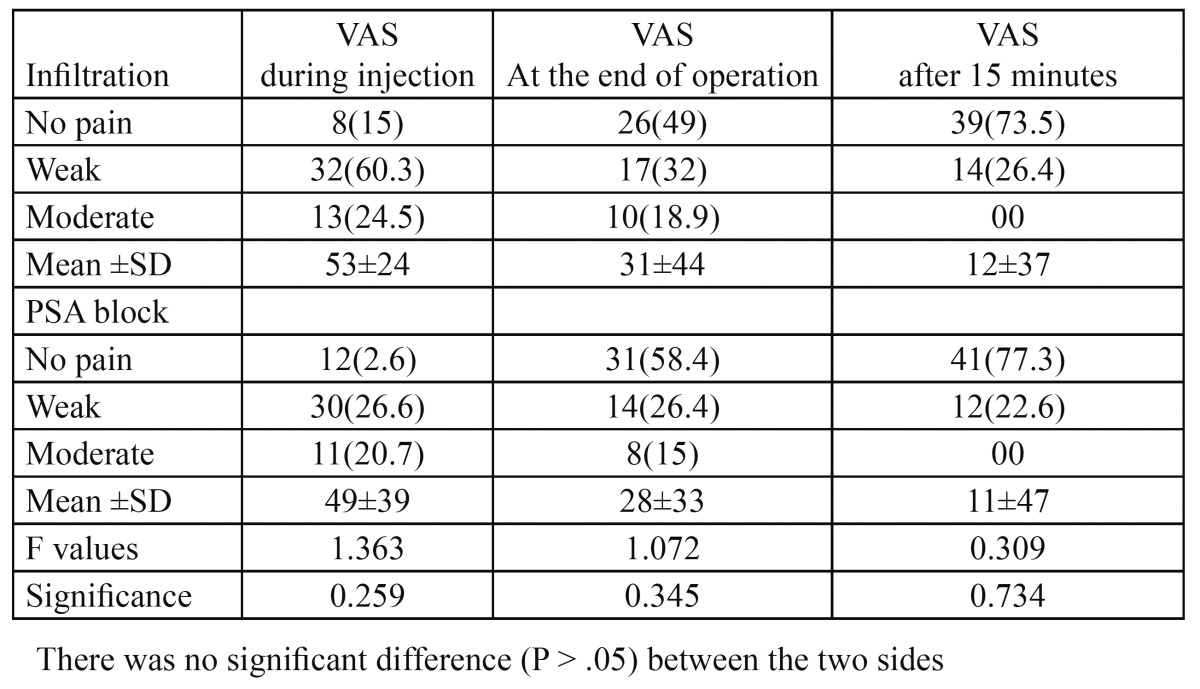
Pain intensity in two sides at different times.

We did not note any significant differences between the PSA side and the infiltrations side in terms of needing for a second or third injection, P=0.096 but little increase in the frequency of repeating injection on the infiltration side was observed.

**Figure 3:**
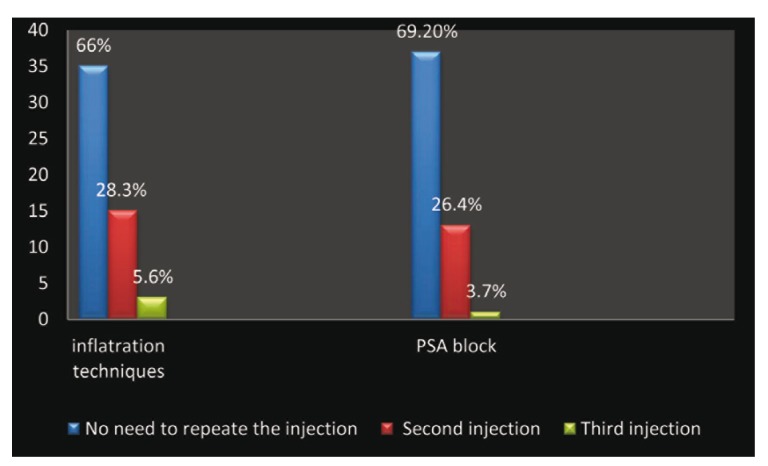
Frequency of needing to repeat the injections.

**Figure 4:**
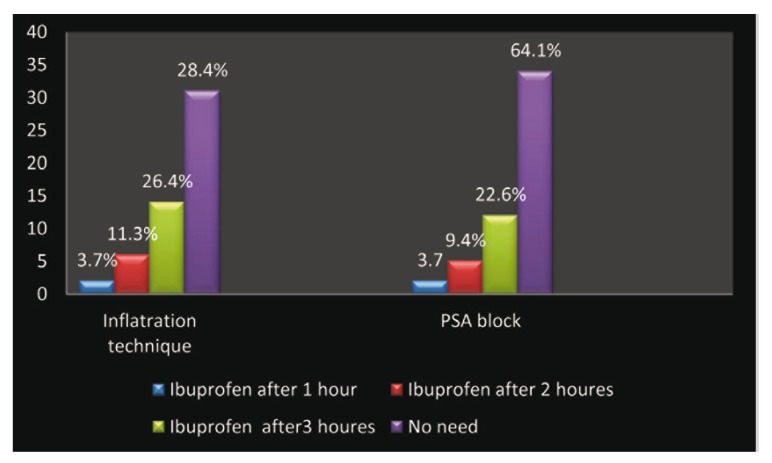
Amount of Ibuprofen required in first three post surgical hours.

The protocol of study also involved assessing NSAID intake in the first three hours. The mean consumption of ibuprofen in number of tablets is noted in Fig. 4. The difference was statistically significant between two techniques at 1, 2 and 3hour intervals post-operatively F= 5.480. Significance =0.020 (p<0.05, Fig. 3).the number of ibuprofen consumption was less in PSA group this because of long duration effect of this technique.

## Discussion

The improvements in agents and techniques for local anesthesia improve the patients` perceptions, comfort and acceptance during dental treatment. The pain control is an important factor for reducing the fear and anxiety associated with dental procedures (12) for that the choice of local anesthetic techniques may influence the amount of discomfort produced during intraoral injection in order to propose an easy and safe method to anesthetize the dentition and surrounding hard and soft tissues during management of surgical extraction (13).

Maxillary infiltration anesthesia is a common method to anesthetize maxillary teeth (14). Also the PSA nerve block has been advocated to anesthetize the first, second, and third molar teeth (11).

In the current study, the success of maxillary PSA block and infiltration technique have been evaluated using the VAS while in the previous studies (15-17) they tried to use the electric pulp tester.

According to the findings of the present comparative study, it can be concluded that there was no difference in the pain experienced by patient using either PSA nerve block technique or infiltrations technique,in surgical extraction of maxillary third molars during the injection or in the post surgical periods ( Table 1, Fig. 2). This came in line with the Padhye et al. study (7) as well as the Aggarwal et al`s study (8).But in the present study, analyzed parameters were related to third molar surgical extraction while the previous mentioned two studies related to conventional normal tooth extraction (7) and irreversible pulpitis (8). The strengths of this study were the consistency of only one surgeon and intra individual evaluation.

There were positive blood aspirations during the PSA in the observation of Pfeil et al. (6), but our resulting data show that the results of this study are that there is positive percentage of 1.8 for PSA and without any positive aspiration in the filtration side. This finding was recorded by others (7,11) in addition to the same finding of non significant differences between the PSA side and the infiltrations side in terms of needing for a second or third injection (Fig. 3).

Preventive strategies for postoperative management of pain and inflammation are based on the known ability of NSAIDs to block the arachidonic acid cascade but concurrent use of NSAIDs should be avoided when possible because of the risk of producing gastrointestinal tract hemorrhage (18). In this study the mean consumption of ibuprofen tablet was highly correlated with PSA block (Fig. 4) because of long duration effect of this technique. And this finding was opposite to the result of Padhye et al. (7).

PSA block was used to overcome the variation in the anatomy of the roots and nerve pathways or even in the presence of infection (19). Some adverse events have been reported with the PSA block including transient diplopia, mydriasis, double vision, and hematomas (20). Hematoma is usually produced by inserting the needle too far posteriorly into the pterygoid plexus of veins. (14) With good technique, hematomas should not be a problem with the PSA nerve block (6).

In conclusion, the statistical analysis of the study results has confirmed clinical equivalence for the surgical extraction of maxillary third molars with PSA nerve block and infiltration technique with the mean advantages of PSA that Minimum number of necessary injections but the risk of a potential complication also must be considered whenever the PSA block is used.
